# The mechanism by which imeglimin inhibits gluconeogenesis in rat liver cells

**DOI:** 10.1002/edm2.211

**Published:** 2021-02-23

**Authors:** Guillaume Vial, Frédéric Lamarche, Cécile Cottet‐Rousselle, Sophie Hallakou‐Bozec, Anne‐Laure Borel, Eric Fontaine

**Affiliations:** ^1^ Université Grenoble Alpes Grenoble France; ^2^ Inserm U 1042 Grenoble France; ^3^ Université Grenoble Alpes LBFA Grenoble France; ^4^ Inserm U 1055 LBFA Grenoble France; ^5^ Poxel SA Lyon France

**Keywords:** complex I, imeglimin, metformin, mitochondria

## Abstract

**Aims:**

To understand the mechanism by which imeglimin (a new oral hypoglycemic agent whose phase 3 development program in Japan has now been completed) decreases hepatic glucose production.

**Materials and methods:**

We compared the effect of imeglimin and metformin on glucose production, ATP/ADP ratio, oxygen consumption rate, mitochondrial redox potential and membrane potential in primary rat hepatocytes.

**Results:**

We found that both imeglimin and metformin dose‐dependently decreased glucose production and the ATP/ADP ratio. Moreover, they both increased mitochondrial redox potential (assessed by mitochondrial NAD(P)H fluorescence) and decreased membrane potential (assessed by TMRM fluorescence). However, contrary to metformin, which inhibits mitochondrial Complex I, imeglimin did not decrease the oxygen consumption rate in intact cells. By measuring the oxygen consumption of in situ respiratory chain as a function of the concentration of NADH, we observed that imeglimin decreased the affinity of NADH for the respiratory chain but did not affect its Vmax (ie competitive inhibition) whereas metformin decreased both the Vmax and the affinity (ie uncompetitive inhibition).

**Conclusions:**

We conclude that imeglimin induces a kinetic constraint on the respiratory chain that does not affect its maximal activity. This kinetic constraint is offset by a decrease in the mitochondrial membrane potential, which induces a thermodynamic constraint on the ATPase responsible for a decrease in the ATP/ADP ratio.

## Graphical abstract

This work specifies the mitochondrial mechanism of action of Imeglimin. It is demonstrated for the first time that an inhibition of the respiratory chain can be competitive.

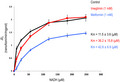

## 1 INTRODUCTION

The mitochondrial respiratory chain is a multi‐protein complex embedded the inner mitochondrial membrane. It catalyses a sequence of redox reactions which couple an electron flux with a vectorial transfer of protons from the matrix to the intermembrane space, thereby generating the mitochondrial membrane potential. The reaction begins at the level of Complex I, a large protein complex with at least 45 subunits, when electrons pass from NADH to ubiquinone. Electrons from the ubiquinone pool then pass through Complexes III and IV to finally reduce oxygen into water. The mitochondrial membrane potential thus established is then used to synthesize ATP from ADP plus phosphate.

Imeglimin is a novel oral antidiabetic agent. Its structure and proposed mechanism of action establish imeglimin as the first in a new class of tetrahydrotriazine‐containing molecules called ‘glimins’. Imeglimin's safety and efficacy as a monotherapy in Caucasian patients with type‐2 diabetes^1^ have been demonstrated. In addition, imeglimin has been shown to be an effective and safe add‐on therapy to metformin and sitagliptin,^2^, ^3^ highlighting its potential as combination therapy with common oral antidiabetic agents. Imeglimin's phase 3 development program in Japan—referred to as TIMES (Trials of imeglimin for Efficacy and Safety)—has now been completed.

Imeglimin has been shown to normalize glucose tolerance and insulin sensitivity in a murine model of type‐2 diabetes by protecting mitochondrial function from oxidative stress and favouring lipid oxidation in the liver.^4^ Imeglimin has also been shown to decrease hepatic glucose production in isolated hepatocytes.^5^ Although imeglimin does not inhibit cell respiration,^6^ indirect evidence suggests that it may unconventionally affect respiratory chain Complex I.^7^

Metformin is the most widely prescribed drug to treat patients affected by type‐2 diabetes and is recommended as a first‐line oral therapy by both American and European guidelines. Metformin is believed to inhibit glucose production because it leads to a mild inhibition of respiratory chain Complex I,^8^, ^9^, ^10^ which decreases ATP production and the ATP/ADP ratio. Although infrequent, lactic acidosis has been reported during metformin poisoning or renal failure due to greater inhibition of Complex I.^11^

Surprisingly, both imeglimin^5^, ^6^ and metformin (^9^ for a review) exhibited antiapoptotic properties. Together, these data suggest that the two drugs may share some common features with respect to mechanism of action. In this work, we compared the effect of imeglimin and metformin on glucose production and tried to clarify the mechanism by which imeglimin inhibits gluconeogenesis.

## 2 MATERIAL AND METHODS

### 2.1 Animals

The 20 male Wistar rats (10–14 weeks old, 330–370 g) used for this study were obtained from Charles River (L’Arbresle, France). The study was carried out in accordance with the European Directives 86/609/EEC, 2010/63/UE on the care, welfare and treatment of animals. All the procedures were reviewed and approved by the ethics committee (Cometh Grenoble) affiliated to the animal facility #D3842110001, and authorized by the French Ministry of Research on 1 December 2017 (APAFIS#9994‐2017052214053756).

### 2.2 Reagents

Imeglimin was from Poxel (Lyon, France). Tetramethyl rhodamine methyl ester (TMRM) was purchased from Molecular Probes (Eugene, OR). Minimum essential medium, M199 medium, phosphate‐buffered saline (PBS) and antibiotics were purchased from Dutscher (Brumath, France). NaCl, KCl, MgSO4, KH2PO4, NaHCO3 and NADH were purchased from Merck (Saint Quentin Fallavier, France). Other reagents were obtained from Sigma (Saint Quentin Fallavier, France).

### 2.3 Isolation and primary culture of rat hepatocytes

Wistar rat liver hepatocytes were isolated from overnight‐starved animals by enzymatic digestion with collagenase according to the method of Berry and Friend^12^ modified by Groen.^13^ Hepatocytes were then seeded on either collagen type I‐coated glass coverslips (for confocal microscopy) or flasks (for metabolic analyses) in a mixture containing 75% minimum essential medium and 25% M199 medium supplemented with 10% foetal bovine serum and antibiotics. The medium was removed after 4 h and replaced by another one, free of serum but supplemented or not with metformin and/or imeglimin (1 mM, unless otherwise specified), and supplemented with glucose (final concentration 3 g/L).

### 2.4 Incubation of rat hepatocytes in condition of glucose production

After an overnight incubation, cells were quickly washed with PBS, to eliminate traces of glucose and then incubated at 37°C for 1 h in a Krebs‐Ringer‐bicarbonate‐calcium buffer free of metformin or imeglimin [120 mM NaCl, 4.8 mM KCl, 1.2 mM KH_2_PO_4_, 1.2 mM MgSO_4_, 24 mM NaHCO_3_ and 1.3 mM CaCl_2_ (pH 7.4)] containing 2 mM sodium pyruvate and 20 mM sodium lactate, saturated with O_2_ : CO_2_ (19:1). The extracellular medium was collected after a 15, 30 then 45 minutes incubation in order to measure the glucose concentration. In parallel experiments, samples of cultured hepatocytes were withdrawn after a 30 min incubation in order to measure phosphate and adenine nucleotide contents, oxygen consumption and the affinity of NADH for the respiratory chain.

### 2.5 Glucose production

The extracellular medium was precipitated with 70% perchloric acid (PCA) to stop the reaction. The lysate was neutralized with a mixture of KOH (2M) and MOPS (300 mM), spun and kept at 4°C until analysis. The glucose concentration was measured enzymatically as described by Bergmeyer^14^ and plotted according to incubation time. The slope of the relationship between the 3 concentrations (determined by computer analysis, Excel^®^) was considered as the rate of glucose production.

### 2.6 Determination of ATP, ADP and AMP content

Hepatocytes withdrawn after 30 min of incubation were lysed in ice‐cold PCA (2.5%)‐EDTA (6.25 mM) for 5 min. The insoluble material was eliminated by centrifugation at 12,000 g for 5 min, and the supernatant fraction was immediately neutralized with KOH/MOPS. After the removal of the formed KClO_4_ by quick spin, the final extract was analysed by HPLC as described by Argaud et al.^15^

### 2.7 Determination of inorganic phosphate content

Hepatocytes withdrawn after 30 min of incubation were quickly washed with saline buffer (NaCl 9 g/L) to eliminate traces of phosphate and then lysed in ice‐cold PCA (2.5%)‐EDTA (6.25 mM) for 5 min. The insoluble material was eliminated by centrifugation at 12,000 g for 5 min, and the supernatant fraction was immediately neutralized with KOH/MOPS. After the removal of the formed KClO_4_ by quick spin, the inorganic phosphate content was measured according to the method of Sumner.^16^ Inorganic phosphate concentration was then calculated assuming a volume of 3.4 × 10^−9^ cm^3^ per hepatocyte^17^.

### 2.8 Measurement of oxygen consumption in intact cells

Hepatocytes withdrawn after 30 min of incubation were transferred into a thermostatically controlled oxygraph vessel equipped with a Clark oxygen electrode. Total as well as rotenone‐sensitive and oligomycin‐sensitive oxygen consumption rates were measured before and after the addition of 2.5 µM rotenone and 2 µM oligomycin, respectively.

### 2.9 Measurement of the affinity of NADH for the respiratory chain

Hepatocytes withdrawn after 30 min of incubation were harvested by centrifugation. Cell pellets were washed with PBS, then immediately resuspended in water for 5 min. The medium was then supplemented with 5 mM Pi (pH 7.4) and transferred into a thermostatically controlled oxygraph vessel equipped with a Clark oxygen electrode. Oxygen consumption rate was measured before and after the addition of increasing concentrations of NADH.

### 2.10 Measurement of NAD(P)H and mitochondrial membrane potential (∆Ψm)

Overnight incubated hepatocytes were studied by time‐lapse laser confocal microscopy at 37°C in a humidified atmosphere (95% air, 5% CO_2_) using a microscope equipped with a perfusion chamber (POC chamber, LaCom^®^, Erbach, Germany) and an incubation system (O_2_‐CO_2_‐°C, PeCom^®^, Erbach, Germany). Hepatocytes washed with PBS were incubated for 30 min in a Krebs‐Ringer‐bicarbonate‐calcium buffer (glucose free and without metformin or imeglimin) containing 2 mM sodium pyruvate, 20 mM sodium lactate and 20 nM TMRM.

Images were collected with a Leica TCS SP2 AOBS inverted laser scanning confocal microscope equipped with a Coherent 351–364 UV laser using a 63X oil immersion objective (HCX PL APO 63.0 × 1.40 W Corr). Laser excitation was 351–364 nm for NAD(P)H and 543 nm for TMRM. Fluorescence emission adjusted with AOBS was 390–486 nm for NAD(P)H and 565–645 nm for TMRM. In order to allow the overlay of NAD(P)H and TMRM signals, image acquisition was set with the same pinhole aperture (Airy 3.55), necessarily increased because of the low signal of NAD(P)H autofluorescence.

Experiments were performed on a randomly chosen field containing 15–25 cells. The background noise of NAD(P)H autofluorescence was removed by fine filter (Kernel 3×3) using Volocity^®^ (Improvision) software, while the other images (TMRM) were not electronically manipulated. Image quantification was performed using ImageJ (NIH images) and Volocity^®^ (Improvision) software as described in Dumas et al.^18^

### 2.11 Statistics

Results are presented as mean ± SEM. The statistical significance of differences was analysed by one‐way ANOVA followed by Tukey‐Kramer HSD post hoc test or by Dunnett's test using JMP^®^ (SAS) software. Significance was defined as *p* < .05.

## 3 RESULTS

**FIGURE 1:**
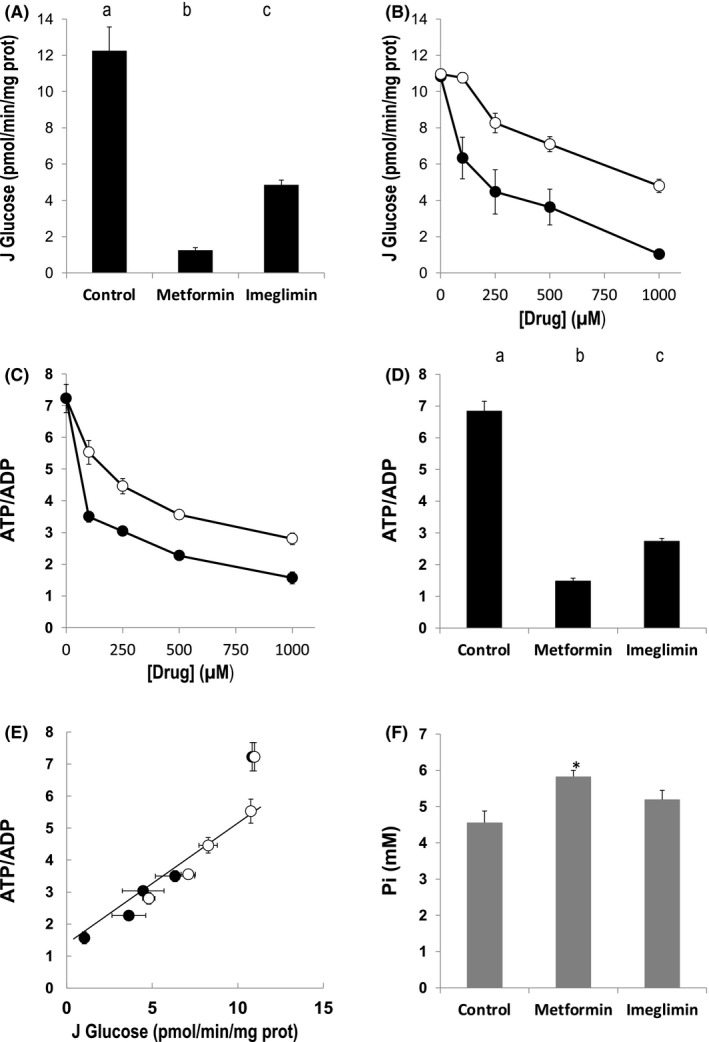
Effect of metformin and imeglimin on glucose production and energy status of primary isolated rat hepatocytes. Hepatocytes incubated overnight in the presence or not of 1 mM metformin or 1 mM imeglimin (Panels A, D and F) or the indicated concentration of metformin or imeglimin (Panels B and C) were washed with PBS and incubated at 37°C for 1 h in a Krebs‐Ringer‐bicarbonate‐calcium buffer (glucose free) containing 2 mM sodium pyruvate and 20 mM sodium lactate, saturated with O_2_ : CO_2_ (19:1). Glucose production, adenine nucleotide and phosphate contents were measured as indicated in Material and Methods. Panel E: data from Panel B and D were plotted. Open circles, imeglimin. Closed circles, metformin. The best correlation (*R* ^2^ > 0.97) calculated by computed analysis (Excel^®^) was obtained when excluding the control condition (data obtained in the absence of metformin or imeglimin). Results are presented as mean ± SEM, *n* = at least 9 (Panel A), 3 (Panel B), at least 6 (Panel C) at least 15 (Panel D) and 5 (Panel F). Values not connected by the same letter in Panel A and B are significantly different (Tukey‐Kramer HSD post hoc test, *p* < .05). Panel F, * significantly different from the proper control (Dunnett's test, *p* < .05)

As expected, results in Figure 1A and B confirm that metformin decreased glucose production in primary rat liver cells. Interestingly, imeglimin also dose‐dependently decreased glucose production, however, to a less marked extent than metformin for each given concentration used.

Because the effect of metformin on gluconeogenesis is attributed to its inhibitory effect on ATP synthesis, we next compared the effect of metformin and imeglimin on the ATP/ADP ratio. As seen in Figure 1C, both drugs dose‐dependently decreased the ATP/ADP ratio in primary rat liver cells. As observed for glucose production, the effect of imeglimin was less marked than that of metformin for each given concentration used (Figure 1C and D). Interestingly, below a threshold value of approximately 6 for the ATP/ADP ratio, glucose production was linearly correlated with the ATP/ADP ratio (see Figure 1E), suggesting that both drugs decreased gluconeogenesis via the same mechanism, that is by decreasing the ATP/ADP ratio.

Thermodynamically, the energy released during ATP consumption depends on the phosphate potential (∆G = ∆G_0_ – RT ln ATP/ADP.Pi), which depends on both the ATP/ADP ratio and phosphate concentration. As shown in Figure 1F, both metformin and imeglimin increased Pi concentration, although the effect was not statistically significant with imeglimin. These data indicate that both metformin and imeglimin decreased the energy charge and thus the free energy released per molecule of ATP hydrolysed.

**FIGURE 2:**
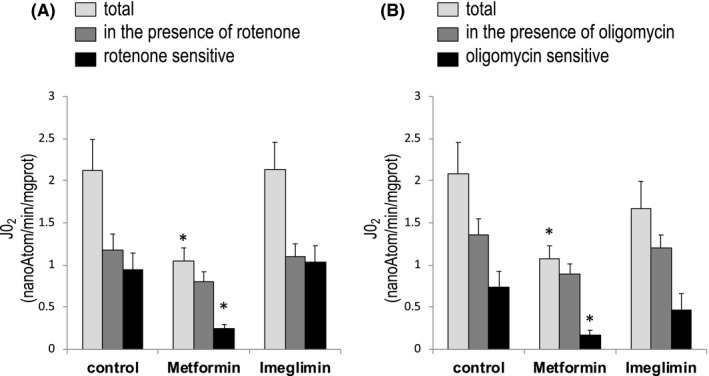
Effect of imeglimin and metformin on the oxygen consumption rate of primary isolated rat hepatocytes. Hepatocytes incubated overnight in the presence or not of 1 mM metformin or 1 mM imeglimin were washed with PBS and incubated at 37°C in a Krebs‐Ringer‐bicarbonate‐calcium buffer (glucose free) containing 2 mM sodium pyruvate and 20 mM sodium lactate, saturated with O_2_ : CO_2_ (19:1). Cells were withdrawn after 30 min of incubation in order to measure their oxygen consumption rate before and after the addition of rotenone or oligomycin. Inhibitor‐sensitive respiration was calculated by subtracting the oxygen consumption after adding the inhibitor to the oxygen consumption before that addition. Results are presented as mean ± SEM, *n* = 6. *Significantly different from the proper control (Dunnett's test, *p* < .05)

Because metformin is known to inhibit mitochondrial chain Complex I, we next tested whether imeglimin affected the ATP/ADP ratio and phosphate potential due to a similar inhibition of the mitochondrial respiratory chain. The oxygen consumption rate of intact cells was then measured before and after the addition of the Complex I inhibitor rotenone, in order to calculate the rotenone‐sensitive respiration (ie the oxygen consumption directly related to Complex I activity). As expected, metformin decreased the rotenone‐sensitive oxygen consumption rate (Figure 2A) whereas imeglimin did not affect either the total or the rotenone‐sensitive oxygen consumption rate of rat liver cells.

We next measured oxygen consumption rates before and after the addition of ATP synthase inhibitor oligomycin, in order to calculate the oligomycin‐sensitive respiration (ie the oxygen consumption directly related to ATP synthesis). As expected, the addition of oligomycin decreased oxygen consumption rates regardless of the condition studied (Figure 2B). However, because metformin decreased the total oxygen consumption (due to Complex I inhibition), ATP synthase activity (the oligomycin‐sensitive respiration) was already low and thus oligomycin had a minimal effect. Interestingly, imeglimin did not affect the oligomycin‐sensitive respiration, indicating that it did not inhibit the ATP synthase activity, either directly or indirectly.

In order to understand the paradox between a decrease in the ATP/ADP ratio in the presence of a normal oxygen consumption rate, we next semi‐quantitatively measured the two forces upstream phosphate potential (ie the mitochondrial membrane potential and the mitochondrial redox potential). The mitochondrial membrane potential in intact cells was assessed by measuring the fluorescence of TMRM probe that accumulates in mitochondria according to the mitochondrial membrane potential, while the mitochondrial redox potential was determined by measuring the autofluorescence of NAD(P)H which is mainly located inside mitochondria. Because the fluorescence measured depends on the number of cells observed, comparisons between the different conditions were performed using fluorescence ratios (NAD(P)H/TMRM) which are not expected to depend on the number of cells.

**FIGURE 3:**
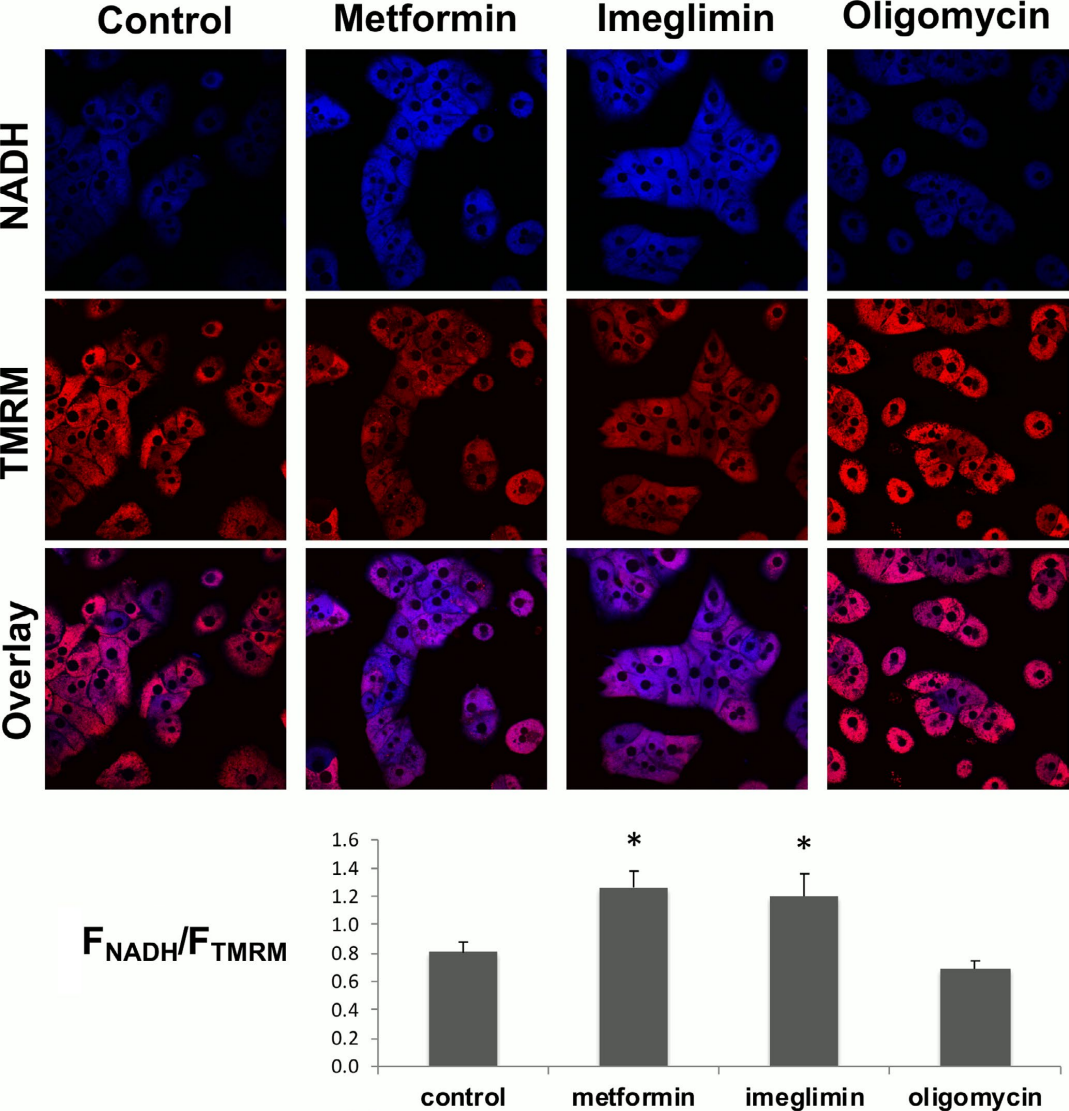
Effects of imeglimin, metformin and oligomycin on NAD(P)H autofluorescence and mitochondrial electrical membrane potential. Hepatocytes incubated overnight in the presence or not of 1 mM metformin or 1 mM imeglimin were washed with PBS and incubated at 37°C for 30 min in a Krebs‐Ringer‐bicarbonate‐calcium buffer (glucose free) containing 2 mM sodium pyruvate and 20 mM sodium lactate, saturated with O_2_ : CO_2_ (19:1) and supplemented with 20 nM TMRM. The fluorescence of NAD(P)H (pseudo blue colour) and TMRM (pseudo red colour) was imaged in randomly chosen fields. When indicated, cells were incubated in the presence of 2 µM oligomycin 5 minutes before imaging. Image quantification was performed as described in Material and Methods. The number of cells observed varying according to experiments, and the fluorescence depending on the number of cells, comparisons were performed by dividing the NAD(P)H fluorescence by the TMRM fluorescence for each field. Results are presented as mean ± SEM, *n* = at least 8. *Significantly different from control (Dunnett's test, *p* < .05)

As expected, metformin, which inhibits Complex I activity, led to an increase and a decrease in NAD(P)H and TMRM signal, respectively, leading to an increase in the NAD(P)H/TMRM ratio (Figure 3). These data are in agreement with previous results showing that metformin decreases the membrane potential^19^, ^20^ and increases the 3‐hydroxybutyrate/acetoacetate ratio,^10^ which is in thermodynamic equilibrium with the mitochondrial redox potential in intact cells. Surprisingly, and despite the fact that it did not inhibit cellular respiration, imeglimin also increased the NAD(P)H/TMRM ratio (Figure 3), suggesting that imeglimin may affect the mitochondrial respiratory chain.

**FIGURE 4:**
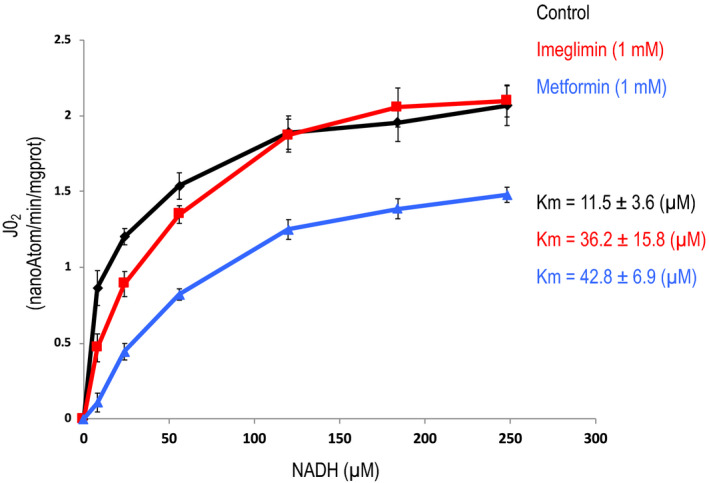
Effects of imeglimin and metformin on the affinity of NADH for the respiratory chain. Hepatocytes incubated overnight in the presence or not of 1 mM metformin or 1 mM imeglimin were washed with PBS and incubated at 37°C for 30 min in a Krebs‐Ringer‐bicarbonate‐calcium buffer (glucose free) containing 2 mM sodium pyruvate and 20 mM sodium lactate, saturated with O_2_ : CO_2_ (19:1). Hepatocytes were harvested by centrifugation and exposed to an osmotic shock (resuspended in water for 5 min) and then incubated in the presence of the indicated concentration of NADH. Rotenone‐sensitive oxygen consumption rate was measured after the addition of Rotenone. Results are presented as mean ± SEM, *n* = 6

In the experiment shown in Figure 4, we then studied the affinity of the respiratory chain for its natural substrate (ie NADH). Because NADH does not freely pass either the plasma membrane or the inner mitochondrial membrane, primary hepatocytes were first exposed to an osmotic shock in order to break the membranes. As a consequence, the mitochondrial activity was measured on uncoupled mitochondria (ie the oxygen consumption rate was not controlled by the membrane potential any longer).

As shown in Figure 4, metformin dramatically decreased the Vmax of the reaction, in agreement with previous results.^8^, ^10^ Moreover, it also affected the affinity of NADH for the respiratory chain, indicating that metformin induced an uncompetitive inhibition of the respiratory chain (presumably at the level of Complex I). On the contrary, imeglimin did not affect the Vmax of this enzymatic activity but decreased the affinity (increased the Km) of NADH for the respiratory chain, indicating that imeglimin induced a competitive inhibition of the respiratory chain.

The precise mechanism by which metformin inhibits Complex I remains a matter of debate. It has been proposed that metformin accumulates inside mitochondria, but two different laboratories using radioactive [^14^C] metformin failed to measure metformin accumulation in mitochondria.^21^, ^22^ As shown in Table S1, we did not observe imeglimin accumulation in mitochondria isolated from hepatocytes in which imeglimin inhibits gluconeogenesis (see Figure 1B).

## 4 DISCUSSION

In this work, we have shown that both imeglimin and metformin decreased glucose production, the ATP/ADP ratio and mitochondrial membrane potential, while they both increased the mitochondrial redox potential. However, contrary to metformin, imeglimin did not decrease the oxygen consumption rate in intact cells.

A decrease in the ATP/ADP ratio can be due to a decrease in ATP production or an increase in ATP consumption. A decrease in ATP production could result from either an inhibition or an uncoupling of oxidative phosphorylation, which are expected to decrease and increase the oxygen consumption rate, respectively. On the contrary, an increase in ATP consumption is expected to increase the oxygen consumption rate. Thus, the observation that imeglimin decreased the ATP/ADP ratio with no effect on the oxygen consumption rate is unusual. Moreover, the fact that imeglimin did not affect either the rotenone‐sensitive or the oligomycin‐sensitive respiration rates strongly suggests that imeglimin did not affect the ATP turnover.

This conclusion raises two questions: (i) how is it possible to reduce the ATP/ADP ratio without reducing the turnover of ATP; (ii) how can a simple decrease in the ATP/ADP ratio affect hepatic glucose production?

There needs to be a force to generate a net flux through an enzymatic pathway. This force, commonly named ∆G (the free energy of reaction) corresponds to the difference between the Gibbs free energy of the substrates and the Gibbs free energy of the products of the enzymatic pathway. However, despite a thermodynamically favourable ∆G (ie a negative ∆G), the net flux through an enzymatic pathway can be modulated by kinetic constraints. Importantly, a change in kinetic constraints does not systematically affect the net flux through an enzymatic pathway. This is typically what occurs during a competitive inhibition of an enzyme when the change in the ∆G (ie an increase in the force) counteracts the increase in the kinetic constraints.

Considering oxidative phosphorylation as a whole, the ∆G of that reaction corresponds to the difference between the Gibbs free energy of NADH and the Gibbs free energy of ATP. By increasing the NAD(P)H fluorescence (Figure 3) and decreasing the ATP/ADP ratio (Figure 1) imeglimin affected the ∆G of the oxidative phosphorylation. But despite a more thermodynamically favourable ΔG, imeglimin did not increase the rate of oxidative phosphorylation (Figure 2), suggesting competitive inhibition in one of the steps of the reaction.

Now considering the oxidative phosphorylation process step by step, the kinetic constraint can theoretically be located either at the respiratory chain or at the ATP synthesis level. The ∆G for the respiratory chain corresponds to the potential span between the redox potential (NADH/NAD^+^) and the membrane potential. The ∆G for the ATP synthase corresponds to the potential span between the membrane potential and the phosphate potential. A kinetic constraint at the respiratory chain level is expected to increase the redox potential and/or to decrease the membrane potential. A kinetic constraint at the ATP synthase level is expected to increase the membrane potential and/or to decrease the phosphate potential.

The fact that imeglimin decreased the membrane potential (Figure 3) suggests that imeglimin did not induce a kinetic constraint at the ATP synthase level. On the contrary, the observation that imeglimin increased NAD(P)H fluorescence and decreased the membrane potential (Figure 3) is consistent with a kinetic constraint at the respiratory chain level. This conclusion is further reinforced by the observation that metformin (which inhibits Complex I) led to the same increase in NAD(P)H fluorescence and decrease in membrane potential. The same observation was also made with rotenone (data not shown).

Interestingly, the result in Figure 4 confirms and completes this proposal by showing that imeglimin led to a decrease in the affinity of NADH for the respiratory chain but did not affect its maximal activity, thus indicating that imeglimin led to a competitive inhibition of the respiratory chain.

We compared metformin and imeglimin at the same concentration (1 mM) which does not correspond to the same degree of inhibition of hepatic glucose production (see Figure 1). Note, however, that 100 µM of metformin is sufficient to inhibit Complex I activity in saturating condition (ie Vmax).^23^, ^24^ The relationship between the concentration of NADH and the autofluorescence of NADH is not linear because due to quenching, the fluorescence of NADH decreases when the concentration of NADH increases above 150 µM.^25^ The observation that Complex I inhibition leads to an increase in autofluorescence of NAD(P)H in intact cells (Figure 3) suggests that the concentration of free NAD(P)H in mitochondria is close to the range of concentrations tested in the experiment depicted in Figure 4.

At present, the mechanism by which imeglimin affects the respiratory chain is not known but does not seem to involve its accumulation in mitochondria (see Table S1). It could be argued that imeglimin is released during the isolation procedure. Note that such a release is even more likely to occur during an osmotic shock which depolarizes the mitochondria, yet the effects of metformin and imeglimin clearly persist despite such a treatment (see Figure 4). This observation does not demonstrate that imeglimin does not accumulate in mitochondria, but demonstrates that once the effect is induced on the respiratory chain, it persists in the absence of imeglimin. Moreover, as observed with metformin,^8^, ^10^ the decrease in glucose production induced by imeglimin required a prolonged incubation of the cells with the drug while direct incubation of isolated mitochondria with imeglimin did not affect the Km of the respiratory chain for NADH (not shown). This suggests that imeglimin (like metformin) affects the respiratory chain via yet unrecognized cell signalling and/or channelling pathways.

Whether imeglimin affects the respiratory chain directly at Complex I vs. at a downstream step(s) is not resolved in this work. Note, however, that we have previously reported that imeglimin inhibits ROS production induced by reverse electron flux through Complex I,^6^ suggesting that imeglimin does affect Complex I either directly or indirectly.

**FIGURE 5:**
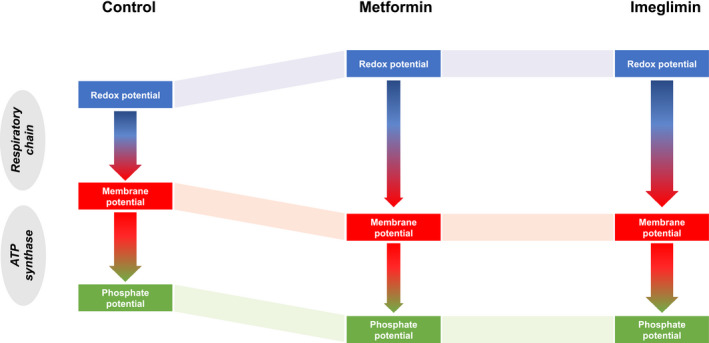
Summary of the results of the respective effects of imegliminin and metformin on flux‐force relationships during oxidative phosphorylation. The redox potential is the driving force used by the mitochondrial respiratory chain to build up and maintain the membrane potential, which in turn is the driving force used by ATPase to convert ADP and phosphate to ATP, thereby creating the phosphate potential. Both imeglimin and metformin increase the redox potential and decrease the membrane potential. Such an increase in the potential span between the forces upstream and downstream the respiratory chain is consistent with a kinetic constraint imposed to the reaction. However, while metformin behaves as a noncompetitive inhibitor, thereby inhibiting the flux (oxygen consumption), imeglimin behaves as a competitive inhibitor and does not affect the flux. Once the membrane potential has decreased, ATP synthesis transiently decreases thereby decreasing phosphate potential, until the potential span at the level of the ATPase is enough to restore a steady‐state flux of ATP synthesis. The scheme summarizes flux‐force relationships in steady‐state conditions, assuming a similar potential span between the membrane potential and the phosphate potential regardless of the experimental conditions. The width of the arrow is proportional to the flux

Because the potential span between the membrane potential and the phosphate potential is the driving force for ATP synthesis, the decrease in membrane potential induced by imeglimin is supposed to decrease ATP synthase activity. As a consequence, ATP production decreases unless phosphate potential also decreases in parallel to achieve the potential span required to sustain a similar rate of ATP synthesis as observed in control conditions. Note that imeglimin did not affect the ATP synthase activity (as assessed by the oligomycin‐sensitive oxygen consumption rate—Figure 2B), but decreased both electrical (Figure 3) and phosphate potentials (Figure 1). We thus propose that the decrease in the ATP/ADP ratio induced by imeglimin is not due to a decrease in ATP synthesis, but on the contrary is a *sine qua non* condition for preservation of a normal rate of ATP synthesis when the membrane potential decreases (Figure 5).

Gluconeogenesis is an ATP‐consuming process controlled by the ATP/ADP ratio.^26^ Indeed, fructose‐biphosphatase is inhibited by AMP,^27^ phosphoenolpyruvate carboxykinase is inhibited by GDP ^28^ while pyruvate carboxylase is inhibited by ADP.^26^ A decrease in the ATP/ADP ratio is thus expected to lead to a decrease in glucose production from lactate.

Because gluconeogenesis from 2 lactates requires 6 ATP to produce 1 glucose, one would expect the inhibition of gluconeogenesis to decrease the ATP turnover. This may not be the case if other ATP‐consuming processes are increased. It is well known that different endergonic reactions do not require the same force to be driven.^29^ Consequently, when phosphate potential decreases, some ATP‐consuming processes become thermodynamically impossible, others slow down, while the less energy‐consuming ones are maintained or even favoured (if controlled by the availability of ATP).

## 5 CONCLUSION

Dissecting the mechanisms by which metformin and imeglimin inhibit gluconeogenesis requires a thermokinetic approach studying the flux‐force relationship. From a thermodynamic point of view, Figure 1 suggests that metformin and imeglimin inhibit gluconeogenesis due to a decrease in its driving force (the ATP/ADP ratio). The difference between the two drugs appears when they are studied from a kinetic point of view. Metformin induces an uncompetitive inhibition of the respiratory chain responsible for a decrease in cellular oxygen consumption rate that may lead to lactic acidosis in case of metformin intoxication. In contrast, imeglimin induces a competitive inhibition of the respiratory chain responsible for the same thermodynamic consequences without affecting the oxygen consumption rate. In other words, the same result is obtained by decreasing both flux and force (metformin) or by decreasing force only (imeglimin). From a theoretical point of view, this remarkable property should minimize the risk of imeglimin inducing lactic acidosis.

## DUALITY OF INTEREST

This work was supported by Poxel SA. No other potential conflicts of interest relevant to this article were reported.

## AUTHORS’ CONTRIBUTIONS

GV and FL contributed to the experimental design, researched data, contributed to the discussion, and review of the manuscript. C. C.‐R. researched data. SH‐B. contributed to the experimental design, discussion and review of the manuscript. A.‐LB contributed to the discussion and review of the manuscript. É.F. contributed to the experimental design, contributed to the discussion and wrote the manuscript. É.F. is the guarantor of this work and, as such, had full access to all the data in the study and takes responsibility for the integrity of the data and the accuracy of the data analysis.

## Supporting information

Supplementary Material
