# Supplementary material for: The mechanism by which imeglimin inhibits gluconeogenesis in rat liver cells
Source: Endocrinol Diabetes Metab. 2021 Feb 23;4(2):e00211. doi: 10.1002/edm2.211 (PMC8029524; doi:10.1002/edm2.211)
Supplement: Supplementary file 1 — Supplementary Material [file EDM2-4-e00211-s001.docx]

**Supplementary data : Intracellular localization of imeglimin**

**Material and methods**

After an overnight incubation in the presence or not of 250 µM imeglimin, cells were quickly washed with PBS, to eliminate traces of imeglimin. Cells were rapidly scrapped with mitochondrial extraction buffer (250 mM sucrose, 20 mM Tris-HCl, 1 mM EGTA, pH 7.4), mechanically broken in a Teflon/glass potter (20 up and down) and centrifuged for 10 min at 800 g (4°C). The supernatant was centrifuged for 10 min at 8000 g (4°C). The pellet was resuspended in the same buffer supplemented with 25% Percoll and centrifuged for 35 min at 100,000g at 4°C in order to purify mitochondria from the mitochondria associated membranes (MAM). The supernatant from the second centrifugation was centrifuged for 60 min at 80,000 g to separate the plasma membrane (pellet) from the cytosol (supernatant). Samples were frozen for subsequent measurement of imeglimin by LC-MS/MS and protein quantification.

**Results**

| Cytosol | Mitochondria | MAM | Plasma membrane |
| --- | --- | --- | --- |
| 123.1  ± 12.5 | 0.70  ± 0.15 | 3.24  ± 0.95 | 7.1  ±2.3 |

**Supplementary Table 1 : Imeglimin quantification (µmol/g proteins) in different cellular compartments.** Subcellular fractionation was performed as described in material and methods. Results are presented as mean ± SEM, n = 5 separate experiments. MAM: Mitochondrial associated membranes.
